# Targeted Anti-Biofilm Therapy: Dissecting Targets in the Biofilm Life Cycle

**DOI:** 10.3390/ph15101253

**Published:** 2022-10-12

**Authors:** Fanqiang Bu, Mengnan Liu, Zixu Xie, Xinyu Chen, Guofeng Li, Xing Wang

**Affiliations:** State Key Laboratory of Organic-Inorganic Composites, Beijing Laboratory of Biomedical Materials, Beijing Advanced Innovation Center for Soft Matter Science and Engineering, College of Life Science and Technology, Beijing University of Chemical Technology, Beijing 100029, China

**Keywords:** biofilm, microenvironment, biofilm-targeting material, antibacterial

## Abstract

Biofilm is a crucial virulence factor for microorganisms that causes chronic infection. After biofilm formation, the bacteria present improve drug tolerance and multifactorial defense mechanisms, which impose significant challenges for the use of antimicrobials. This indicates the urgent need for new targeted technologies and emerging therapeutic strategies. In this review, we focus on the current biofilm-targeting strategies and those under development, including targeting persistent cells, quorum quenching, and phage therapy. We emphasize biofilm-targeting technologies that are supported by blocking the biofilm life cycle, providing a theoretical basis for design of targeting technology that disrupts the biofilm and promotes practical application of antibacterial materials.

## 1. Introduction

Biofilms are still considered as a major cause of chronic infections (such as chronic periapical periodontitis, chronic lung infection, infective endocarditis, etc.) [1,2,3,4,5]. Additionally, biofilms cause extensive damage to the marine environment and agriculture [6,7,8,9,10]. There has thus been considerable interest in the biofilm formation mechanism [11,12,13,14,15,16,17]. Accumulating evidence suggests that these bacterial resistance phenomena result from the ability of bacteria to enter into a dormant or persistent state in the biofilm [18,19,20,21,22,23]. The biofilm forms a complex microenvironment and spatial organization structure, such as extreme internal environments and extracellular polymeric substances (EPS), which limit entry of most drugs into the biofilm [24,25,26,27,28,29,30]. As such, understanding biofilm formation processes and chronic infections that can benefit from changing treatment, and, thus, tailoring personalized treatment to clinical patients, is paramount in improving the anti-biofilm therapeutic efficacy. Nonetheless, clinical treatment protocols for biofilm infections have not been updated accordingly.

**Figure 1:**
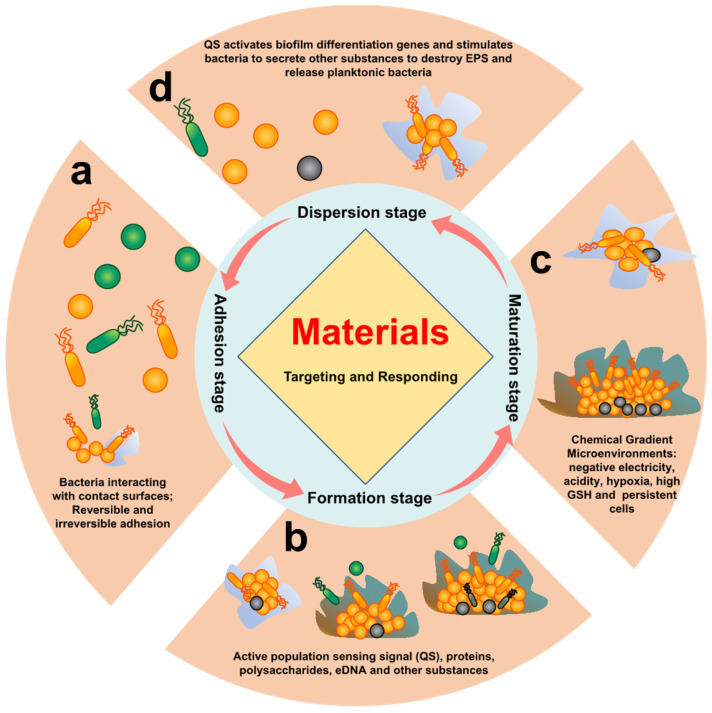
**The biofilm life cycle.** Different stages of bacterial biofilm formation. (**a**) Initial adhesion, in which bacteria adhere to surface of tissue through their own adhesins of bacteria; (**b**) early biofilm development stage, whereby the bacteria begin to divide and produce EPS by quorum sensing, eDNA, polysaccharide, and protein; (**c**) biofilm maturation stage, in which the biofilm will form a stable 3D structure through EPS, and the internal microenvironment exhibits a certain chemical gradient, such as acidity, hydrogen peroxide (H_2_O_2_), hypoxia, and overexpressed glutathione (GSH); (**d**) biofilm dispersion stage, whereby bacteria are oppressed by the extreme microenvironment, and their own secreted enzymes and D-type amino acids lyse the biofilm and return to the state of planktonic bacteria.

The anti-biofilm strategy was still in an early stage of physical clearance and high-dose continuous administration in early clinical studies [31,32]. At present, most biofilm removal methods or treatment methods approved by the US Food and Drug Administration (FDA) focus on retained medical devices [33]. Research has shown that killing bacteria does not necessarily eradicate biofilms. Therefore, the challenge of residual biofilm, which may trigger chronic infections, must be addressed. A comprehensive understanding of the mechanisms and inherent properties of the biofilm life cycle was required to address this grand challenge (Figure 1). The following four stages can accurately represent the process from biofilm formation to re-spreading:

1. Initial adhesion stage: The reversible adhesions are dominated by Lewis acid–base, van der Waals forces, electrostatic interactions, and hydrophilic–hydrophobic interactions [34,35]. Irreversible adhesion is triggered by the bacteria’s own adhesins and adhesion proteins [36]. Reversible and irreversible adhesion of bacteria to the surface is the main feature of this stage.
2. Early biofilm formation stage: After bacteria adhere to the surface, bacteria activate their own metabolic pathways, which induces the bacteria to secrete metabolites (proteins, polysaccharides, eDNA etc.) to form EPS. At the same time, this also promotes bacteria-to-bacteria adhesion and activates quorum sensing (QS) [37]. Proteins, polysaccharides, eDNA, and QS of bacteria are the main features of this stage.
3. Biofilm maturation stage: A complex spatial structure and a microenvironment with chemical gradients (acidity, hypoxia, high reduction, etc.) are gradually formed with the increase in EPS synthesized by bacteria. At the same time, some bacteria will enter a dormant and persistent state [38]. Therefore, the characteristics of this stage are mainly complex chemical gradient microenvironment, persistent cells, and dormant cells.
4. Biofilm dispersion stage: Bacteria will secrete relevant secretions (enzymes, D-amino acids, surfactants, and other substances) to destroy EPS in response to nutrient deficiencies and accumulation of toxic substances, returning to a planktonic state [39]. This stage is characterized by associated secretions of bacteria and residual biofilm after dispersal.

Depending on the growth environment, biofilm formation changes, resulting in different biofilm spatial structures and bacterial gene expression differences [40,41,42,43]. Although the “characteristics” of biofilm have been revealed for many years, the clinical treatment of biofilm infections has not been updated due to the high complexity of biofilms [44,45]. These days, with the rapid growth experienced in materials science, surface-coating and eluting substrates materials are gradually being used clinically to remove biofilms (e.g., antibiotic-loaded bone cement to prevent orthopedic infections) [46,47]. Similarly, studies of biomimicry, surface textures, and chemicals in plants and animals are also promising approaches to preventing microbial adhesion and biofilm formation [48,49]. Using the amino acids and enzymes produced by bacteria to accelerate disintegration of biofilms is also one of the frontiers of anti-biofilm research [50,51]. These materials and technologies are very promising to solve the problem of biofilm infection. Although these studies have some statistical significance, to determine whether these technologies have the potential of being transformed into clinical technologies, researchers have to consider using in vivo or human cell models for further verification. The main reason for this is that most biofilm models are constructed from a single strain in the laboratory, but actual clinical situations may consist of multiple strains or lurking beneath probiotics [52,53]. Relevant studies have pointed out that some strains cannot form biofilm alone, but a variety of strains will help each other to build shelters together [54], such as *Actinomyces naeslundii T14V* and *Streptococcus oralis* (*S. oralis*) *34*, which promote symbiosis in saliva to form biofilm [55]. Compared with a biofilm of a single bacterial strain, the harm of a multi-species biofilm to the host will increase exponentially. Interestingly, bacteria will also have hostile relations that try to destroy the enemy’s shelter [56], such as *Pseudoalteromonas tunicate*, in the process of biofilm formation, which could inhibit and destroy the biofilm formed by other bacteria [57]. Therefore, the mutual hostility or mutual support in vivo between reference bacteria can provide effective theoretical support for design and development of biofilm-targeting materials.

Several excellent reviews discuss the protective mechanisms of biofilms against bacteria in response to antibiotics, antibacterial agents, and host immunity [17,58,59,60]. This review focuses on development and design of specific targeted biofilm therapeutic strategies and materials, as well as the challenges faced. A comprehensive description of how the properties of membranes at different stages can be exploited to design targeted materials, current insights into the targeting of EPS matrices, inhibition of chemical gradients and diffusion pathways, etc., as well as drug resistance and tolerance reversal strategies for dormant cells and persistent bacteria in biofilms, are provided. Furthermore, this paper reviews strategies that are expected to improve the efficacy of current clinical treatment modalities or provide new biofilm-targeting technologies, including the targeting adhesin strategy, quorum quenching, phage-targeting strategy, and targeting dormant cells strategy. Herein, we focus on biofilm-specific targeting materials that can be applied clinically. However, not all biofilm-targeting technologies are limited to clinical but also include agriculture, forestry, marine, and other directions. Therefore, we provide a comprehensive list of recent and prospective biofilm targets in Table 1. Finally, we believe that treatment of mature biofilm infections is more similar to treatment of cancer because their micro-environments are extremely similar (such as low pH, oxygen deficiency, overexpression of GSH, abnormal osmotic pressure, etc.) [17,37,38]. However, it may be more difficult than cancer therapy because the life cycle of biofilm is faster and more uncontrollable. It is important to note that more complex tissue structures, such as EPS, QS, and eDNA, are present in biofilms to inhibit therapeutic effects. More importantly, biofilm infections often exist in complex flora, making it more challenging to specifically target pathogen biofilms and achieve clearance [61,62]. Therefore, in the face of various challenges, biofilm-targeting technology has irreplaceable significance.

## 2. Strategies of Targeting Initial Adhesion Stage

### 2.1. Inhibit Biofilm Formation

Although, most of the time, points studied on biofilms were in the middle and late stages of the biofilm life cycle, we believe that precise targeting of adhesion properties during the first stage of biofilm formation is an effective strategy [63]. First, its advantage is avoiding drug resistance, tolerance, impermeability, etc., caused by the middle and late stages of biofilms. Second, early anti-adhesion strategies can not only effectively inhibit formation of biofilms but also achieve preventive effects.

#### 2.1.1. Targeted Adhesin Strategy

**Figure 2:**
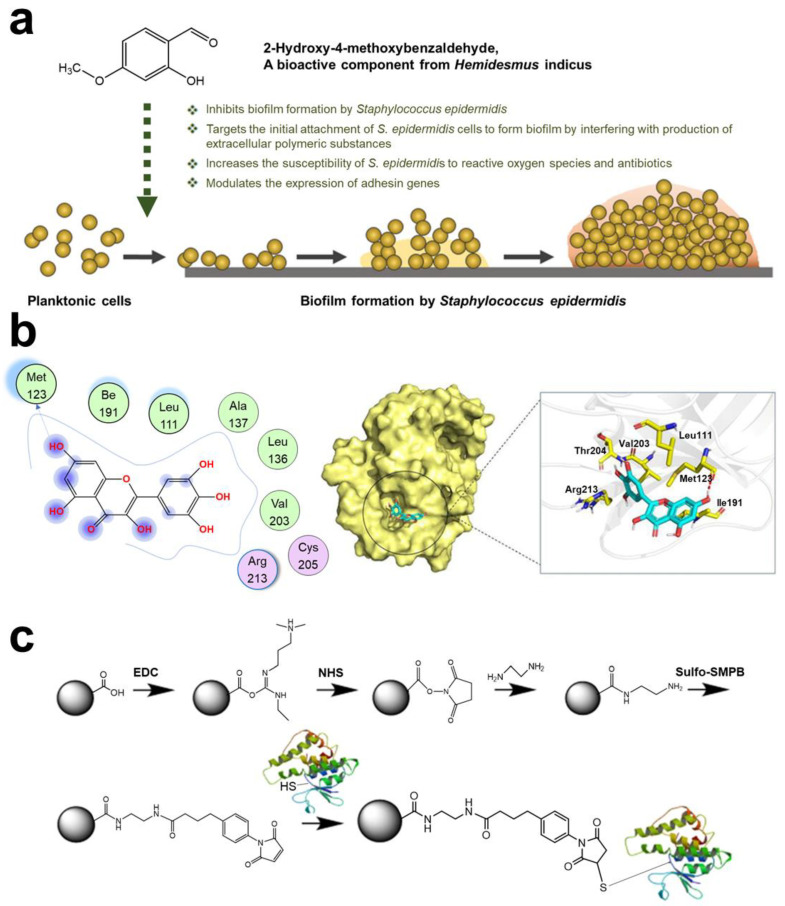
**Schematic diagram of targeting adhesin strategy.** (**a**) Mechanism of HMB targeting *S.epidermidis*. Reprinted with permission from Ref. [73]. Copyright 2020, Arumugam Veera Ravi. (**b**) Action sites of myricetin and *SrtA*. Reprinted from Ref. [74]. (**c**) Interaction mechanism between inhibitor MAM7 and Glutathione-S-Transferase (GST) fusion protein. Reprinted from Ref. [77].

Bacterial adhesin plays a key role in bacterial colonization and subsequent infection. Adhesin or adhesion protein could be used as a bacteria–host cell or bacteria–bacteria “bridge”. Multiple adhesions are activated and expressed (such as proteins, lipids, and glycopolymers) [64]. Moreover, vitronectin and fibrinogen were also used similarly to adhesin [65]. Linke et al. found that *Yersinia enterocolitica* uses tiny sticky hairs to attach to the target. *Yersinia* adhesin A (*YadA*) protein of bacteria could penetrate two layers of cell membrane without any cell energy [66]. Recent research found that targeting-adhesin strategies were not thought to increase bacterial resistance nor interfere with the bacterial life cycle [67,68]. Therefore, the adhesion stage was a strategic step for bacteria, and anti-adhesion therapy could effectively hinder the infection process. Notably, using materials to reduce bacterial adhesion could also promote the host immune system [69,70]. According to these principles, various therapies have been designed. Research by Heras et al. showed that blocking the super adhesion protein (*UpaB*) of bacteria could effectively inhibit bacterial colonization in the host [71]. Zhan et al. synthesized an indole derivative of selenium-containing (SYG-180-2-2, C_21_H_16_N_2_OSe), which could inhibit biofilm by downregulating *icaA* and *icaD* and upregulating *icaR* and *coY*, thereby affecting PIA (*ica*: intercellular adhesin) [72]. Ravi et al. demonstrated that 2-hydroxy-4-methoxyben- zaldehyde (HMB, natural product of *Hemidesmus indicus*) could target the initial cell adhesion of *Staphylococcus epidermidis* (*S. epidermidis*) (Figure 2a) [73]. *Sortase* A (*SrtA)* is able to catalyze the initial adhesion between *Streptococcus mutans* (*S. mutans*) surface protein Pac and lectin. Ma et al. found that myricetin can effectively inhibit *SrtA* (Figure 2b) [74]. Liu et al. confirmed that nucleotide second messengers (such as cyclic adenosine monophosphate (cAMP) and cyclic diguanylate (c-di-GMP)) play an important role in regulating biofilm maintenance. It has been reported that pathogenic bacteria have evolved strategies to manipulate host cAMP concentrations [75]. This discovery provided an important direction for new drug design. Ashraf et al. found that the extract of *Eruca sativa Miller* (*E. sativa*) could effectively target adhesion proteins. A molecular docking analysis of *E. sativa* phytochemicals showed interaction with active site of adhesion proteins *Sortase A*, *EspA*, *OprD*, and *type IV b pilin* of *Staphylococcus aureus* (*S. aureus*), *Escherichia coli* (*E. coli*), *Pseudomonas aeruginosa* (*P. aeruginosa*), and *Salmonella enterica serovar Typhi* (*S. enterica ser. typhi*), respectively [76]. Krachiler et al. designed a functionalized multivalent adhesion molecules (MAM7) adhesive polymer bead that could effectively reduce infection of *P. aeruginosa* in the burn model and promote healing (Figure 2c) [77]. Cardoso et al. used gluconamide moieties to specifically target lipopolysaccharide (LPS) molecules in the outer membrane of *E. coli*, which efficiently prevented non-specific protein adhesion [78].

#### 2.1.2. Interference Adhesion Strategy

**Figure 3:**
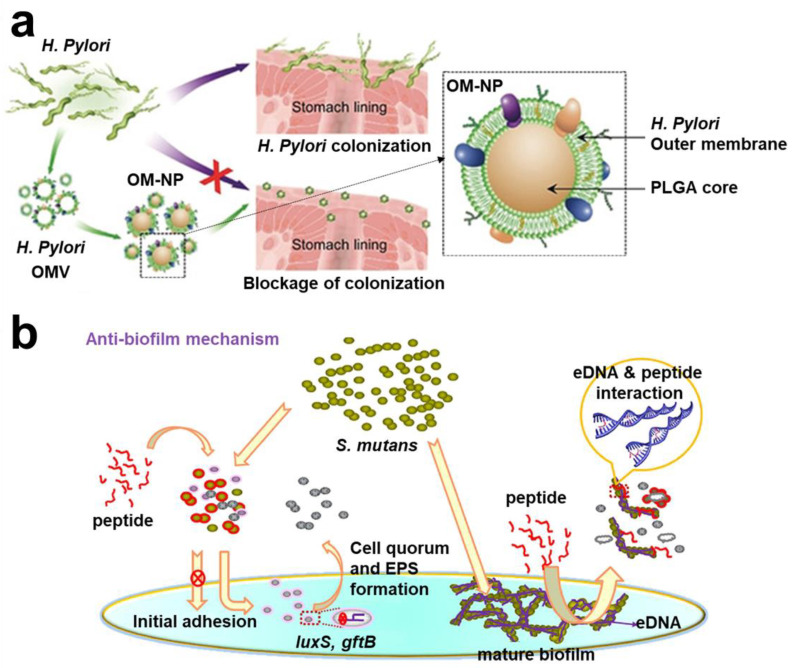
**Schematic diagram of interfering with bacterial adhesion strategies.** (**a**) OM-NPs coated with outer membrane of *H. pylori*, which could compete with bacteria for binding sites on cells. Reprinted with permission from Ref. [86]. Copyright 2012, Prof. Dr. Anke Kruege. (**b**) G3 inhibited bacterial adhesion and interfered with biofilm formation. Reprinted with permission from Ref. [89]. Copyright 2020, Hai Xu.

Developing adhesion targeting compounds has been a long and in-depth development process. Thus, some scholars have proposed a strategy to interfere with adhesin, which uses compounds as analogues of bacterial adhesin receptors to make bacteria “mistakenly” adhere to the host to achieve anti-adhesion effects [79,80]. A treatment scheme using α-mannoside that interferes with FimH1 for treatment of catheter-related urinary tract infection (CAUTI) was applied [81,82]. In addition, Hartmann et al. used mannose-modified diamond to effectively enrich *E. coli* in sewage, achieving the removal effect [83]. In addition, after the discovery of *PapG* protein, a therapeutic scheme to inhibit *PagG* adhesion with galacto-oligosaccharide was finally formed in the clinical environment [84]. Most pathogens were opportunistic pathogens, and interaction of specific receptors and outer membrane molecules between bacteria and tissue cells was a prerequisite for infection [85]. Thus, interfering with bacterial adhesion is a therapeutic strategy that deserves further investigation. Zhang et al. prepared a nanoparticle coated with the outer membrane of *Helicobacter pylori* (*H. pylori*) (Figure 3a). NPs could compete with bacteria for binding sites on cells and inhibit bacteria from adhering to gastric epithelial cells and stomach tissues [86]. L. Davies et al. identified a 20 kDa peptide binding domain in the 1.5 MDa RTX adhesin of marine bacteria (*Vibrio cholerae* and *Aeromonas veronii*). Researchers used peptide library analysis to obtain a tripeptide that could effectively inhibit pathogen adhesion to the host [87]. Choi et al. found that D-arabinose could inhibit biofilm formation of oral bacteria (*S. oralis*, *Fusobacterium nucleatum* (*F. nucleatum*), and *Porphyromonas gingivalis* (*P. gingivalis*)) and the activity of autoinducer 2 (a QS molecule) [88]. Xu et al. designed G(IIKK)_3_I-NH_2_ (G3) based on α- A helical peptide, which inhibited bacterial adhesion and interfered with biofilm formation (Figure 3b) [89].

#### 2.1.3. Surface Anti-Adhesion Strategies

**Figure 4:**
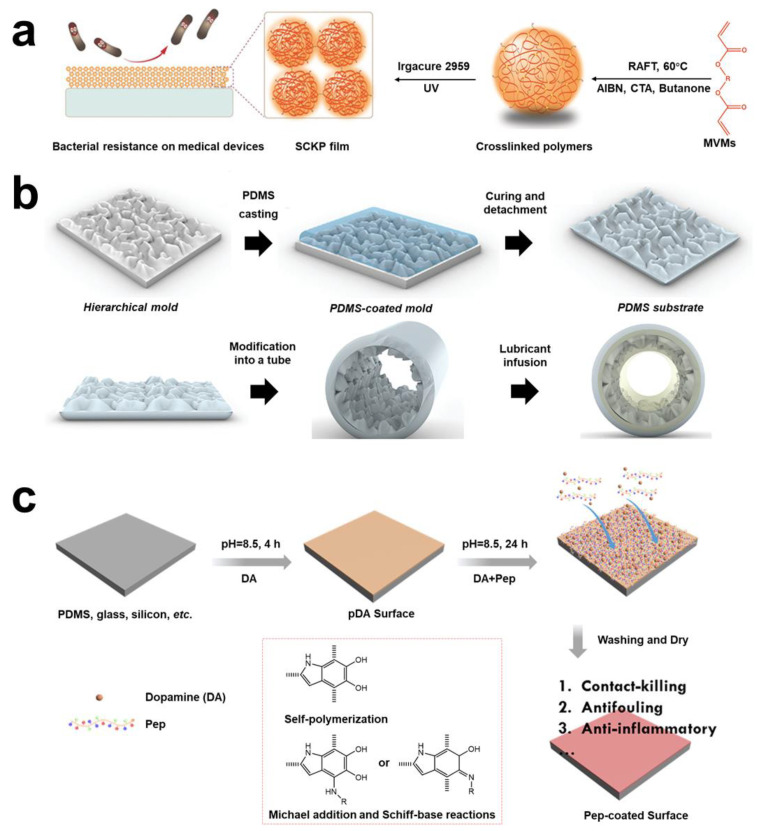
**Schematic diagram of surface anti-adhesion technology.** (**a**) The anti-adhesion polymers were synthesized by RAFT homopolymerization of MVMs. Reprinted with permission from Ref. [91]. Copyright 2019, Prof. Wenxin Wang. (**b**) Transferring the topography present of hierarchical polystyrene surfaces onto PDMS, forming an anti-adhesion, preventing thrombosis, and flexible biocompatible elastomer. Reprinted with permission from Ref. [96]. Copyright 2022, Tohid F. Didar. (**c**) The catechol, cationic, and anionic units to construct a multifunctional modified surface multifunctional coating (mPep) in medical catheters. Reprinted with permission from Ref. [98]. Copyright 2020, Jian Ji.

In clinical practice, biofilm infections caused by implants and medical devices often occur. A foreign body implantation is one of the main causes of biofilm infection [44]. To eliminate this biofilm-related infection, only uninfected medical equipment can be used, along with high-dose antibiotic treatment. Shortly afterwards, Khoo and Ji et al. proposed that endowing anti-adhesion performance to medical devices can better inhibit formation of biofilm and greatly reduce the use of antibiotics [46,90]. Based on this theory, a large number of laboratory designs have been proposed. Based on the optimization strategy of film surface morphology and hydrophobicity, Wang et al. designed four membranes with very high antiseptic properties (Figure 4a) [91]. Inspired by hydration ability of zwitterionic brushes, Hong et al. grafted 2-methacryloyloxyethyl phosphate choline (MPC) onto medical devices, which can effectively inhibit formation of biofilm [92]. Wang et al. proposed a stereochemical antibacterial strategy to achieve an anti-adhesion effect through the selective differentiation of L/D molecules by bacteria [93]. Antognazza et al. patterned silk film substrates that could effectively reduce adhesion of bacteria [94]. Leu et al. modified the polypropylene (PP) surface by reactive ion etching (RIE) technology and reduced the adhesion of *E. coli* on the PP surface, which decreased by 99.6% via pro-hydrophobic interactions [95]. In vitro surface anti-adhesion technology alone does not meet practical clinical needs; it is also vital to address how to apply these techniques in vivo. Didar et al. transferred the topography present with hierarchical polystyrene surfaces onto polydimethylsiloxane (PDMS), which prevents biofilm and thrombosis in vivo (Figure 4b) [96]. Sun et al. integrated highly antibacterial copper nanoparticles (CuNPs) into hydrophilic polydopamine (PDA) coating and finally fixed it on a reverse osmosis (RO) thin-film composite membrane, which could reduce bacterial adhesion and significantly inhibited the formation of biofilm [97]. Ji et al. constructed a multifunctional modified surface multifunctional coating (mPep). Application of mPep in medical catheters in vivo proved to be effective in reducing bacterial adhesion and antibacterial (Figure 4c) [98].

On balance, anti-adhesion technology has a “prevention preferable to treatment” advantage in anti-biofilm infection, and it is also one of the essential conditions for food packaging materials and biological storage materials. The potential advantages of targeted adhesion technology as a vaccine or drug remains underexplored in anti-biofilm therapy. Most compounds of targeting adhesins were easily ignored thus far because they did not exhibit specific minimum inhibitory concentration (MIC) and minimum bactericidal concentration (MBC). Identifying their potential to trigger biological function and effect, in comparison with the main studies, such as those involving peptides and antibiotics, would be of value. Therefore, whether in vivo or in vitro, anti-adhesion technology is very promising to achieve clinical transformation.

### 2.2. Targeting Biofilm Formation Strategy

As planktonic bacteria adhere to tissues or abiotic surfaces, bacteria spontaneously enter the second stage: biofilm formation. At this stage, the bacterial phenotype gradually changes, which causes the bacteria to have mutual adhesion and aggregate, forming small colonies. Bacteria will trigger QS for “communication” during formation of small colonies. Acyl-homoserine lactones (AHL) and autoinducing peptides (AIPs) are signaling molecules that mediate Gram-negative and Gram-positive bacteria, respectively [99]. At the same time, there was a “general language” autoinducer-2 (AI-2) that can mediate both Gram-negative and Gram-positive bacteria [100]. After bacteria receive QS signal molecules, bacteria gradually change their metabolism and participate in biofilm formation, including expression of PIA, bacterial autolysis and death, release of eDNA, and secretion of polysaccharides and proteins [101,102,103]. Finally, EPS is formed under the joint action of various mechanisms [30]. Therefore, the biological behaviors of the above bacteria can be used as potential targets to provide a theoretical basis for design of targeting materials.

#### 2.2.1. Quorum Quenching

Formation of biofilm is a complex and relatively slow dynamic process. QS is the communication language of bacteria, which can effectively tell bacteria what to do now. At present, it is known that QS molecules could directly regulate bacterial behavior in biofilm. Many studies have reported that some compounds had the ability to quench QS, thereby destroying the biofilm, termed quorum quenching (QQ) [104]. These molecules are called quorum sensing inhibitors (QSIs) [105]. QSIs have been found to destroy the QS process mainly through the following ways thus far: 1. inhibit QS molecular synthesis; 2. simulate QS molecules; 3. degrade QS molecules; 4. chemically modify QS molecules. QSI will not affect DNA and cell division of bacteria, so bacteria rarely develop related drug resistance [106]. Many QSI compounds have been found and synthesized now; therefore, this paper only reviews QSI compounds with targeting functions.

**Figure 5:**
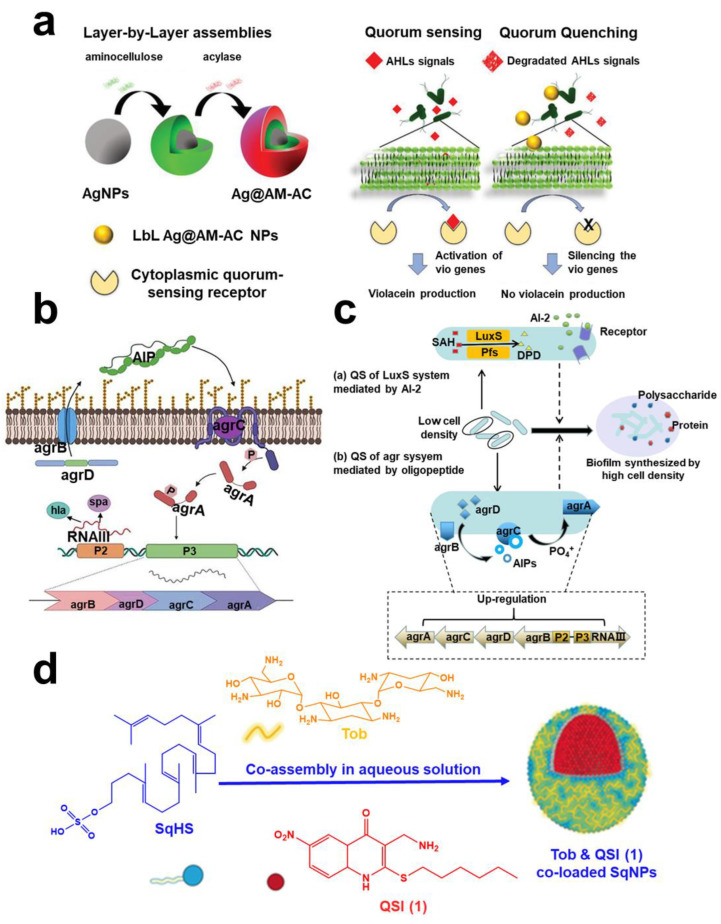
**Quorum sensing targeting technology**. (**a**) A schematic diagram of QQE acylase and amino-bearing biopolymer AM was covered layer by layer on the AgNPs template. Reprinted from Ref. [111]. (**b**) Schematic diagram of the *agr* QS system and expression of QS-related genes in *Methicillin-resistant Staphylococcus aureus* (*MRSA*). Reprinted with permission from Ref. [114]. Copyright 2022, Feng Xu. (**c**) Two quorum sensing systems (a) *LuxS* system and (b) *agr* system in *L. monocytogenes* could be used as targets of quercetin. Reprinted with permission from Ref. [119]. Copyright 2020, Yong Hong Meng. (**d**) The self-assembling nanoparticles of a squalenyl hydrogen sulfate (SqNPs) composed of a new lipophilic QSI (1), tobramycin, and SqHS. Reprinted from Ref. [121].

QSI molecules with targeting function mainly have two mechanisms of action: the first is to target QS synthetase to inactivate or degrade QS signal molecules [107,108]. The second is the receptor that targeted QS signaling molecules so that the receptor cannot receive QS molecules or compete with QS molecules [109,110]. The quorum-quenching enzyme (QQE), such as acylase and lactonase, can degrade the QS signal and destroy QS in the extracellular environment. Tzanov et al. found that QQE acyltransferase could reduce the AHL signal (Figure 5a) [111]. The aceleacin A acylase (*Au* AAC) and N-acyl homoserine lactone acyltransferase (*Au* AHLA) have the same effect [112]. These enzymes have a QS targeting function. In addition, accessory gene regulator (*agr*) is the most classic QS system of *S. aureus* [113]. Xu et al. verified that hyperbranched poly-L-lysine (HBPL) inhibited QS mediated by the *agr* system and inhibited expression of QS-related genes (Figure 5b) [114]. Luteolin, as a QSI, also inhibited downregulation of *agrA* gene, but whether it has a targeted effect needs further study [115]. Bendary et al. further proved that zinc oxide nanoparticles (ZnO NPs), Hamamelis tannin (HAM), and protease K could be used as QSIs to downregulate the *agrA* gene, thereby inhibiting formation of biofilm [116]. Pseudomonas quinolone signal (PQS) is bound by cytosolic LysR-type receptor *PqsR* (also known as *MvfR*) [117]. Therefore, *PqsR* antagonists were found [118]. Recent studies have found that quercetin can specifically target the *lasIR* and *rhlIR* systems of *P. aeruginosa* and *LuxS* and *agr* systems of *Listeria monocytogenes* (*L. monocytogenes*), thereby inhibiting the QS system (Figure 5c) [119,120]. Ho et al. found a new lipophilic QSI for destroying biofilm (Figure 5d) [121].

#### 2.2.2. Targeted Polysaccharide Strategy

**Figure 6:**
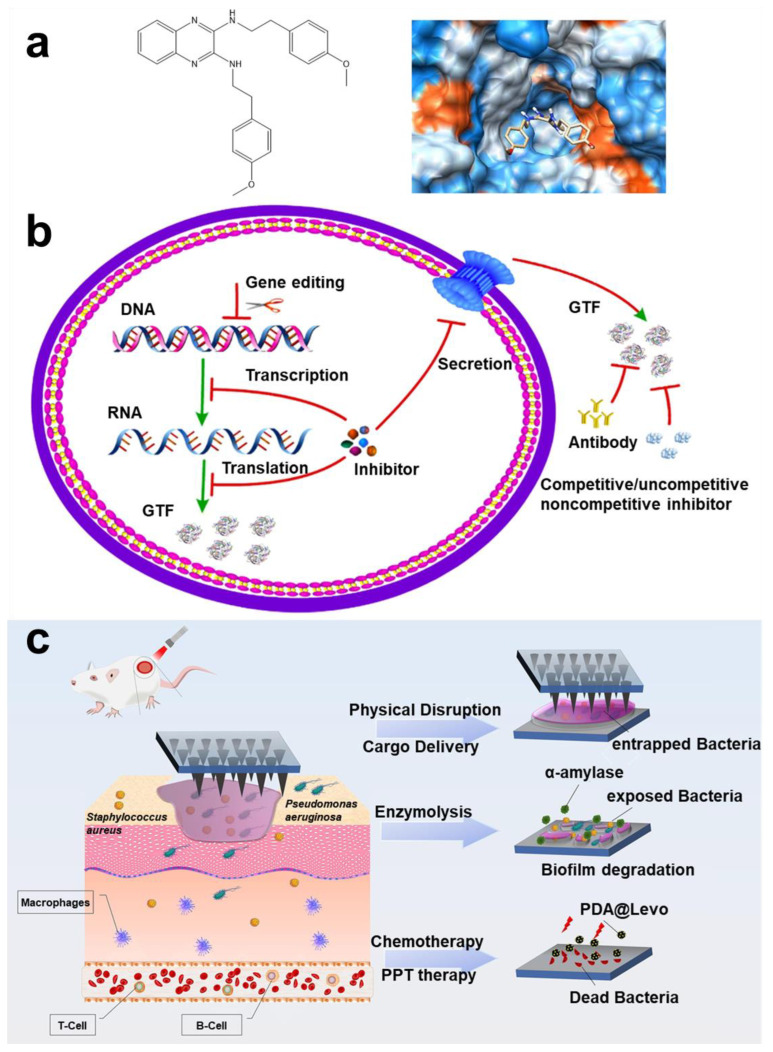
**Polysaccharide targeting technology**. (**a**) 2-(4-methoxyphenyl)-N-(3-{[2-(4-methoxyphenyl) ethyl] imino}-1,4-dihydro-2-quinoxalinylidene) ethanamine targeting glucosyltransferase and docking analysis. Reprinted with permission from Ref. [126]. Copyright 2015, Yuqing Li. (**b**) Schematic diagram of inhibition process of *Gtfs* inhibitors for *S. mutans.* Reprinted from Ref. [135]. (**c**) Schematic diagram of α -amylase-PDA@Levo microneedle patch treating wound biofilm infection in mice. Reprinted with permission from Ref. [135]. Copyright 2022, Daidi Fan.

As one of the important components of the protective barrier and biofilm surface, polysaccharides can enhance intercellular adhesion and aggregation of bacteria, promote bacterial immune escape, stabilize, and maintain the biofilm microenvironment, and provide nutrients for bacteria [122,123,124,125]. Targeted design of related materials and strategies to target polysaccharides in biofilm are effective methods to remove biofilm. The initial targeting strategy is to inhibit enzymes that produce polysaccharides in bacteria, such as *glucosyltransferases* (*Gtfs*) in Gram-positive *S. mutans* and aggregative exopolysaccharides *Psl* and *Pel* in Gram-negative *P. aeruginosa* (Figure 6a) [126,127,128]. At present, development of *Gtfs* inhibitors for *S. mutans* is very extensive (Figure 6b) [129,130]. It is worth noting that *Gtfs* inhibitors are also used in developing vaccines [131]. Similarly, the combination of *Gtfs* inhibitors and drugs could demonstrate practical anti-cariogenic efficacy. *Disperse* B (*DspB*), glycoside hydrolase, and monoclonal antibodies are also common mainstream strategies for targeting EPS. Drug delivery systems (DDS) could protect enzymes from the external environment, and enzymes provide DDS targeting specificity [132]. *DspB* can efficiently and specifically hydrolyze poly-beta (1,6)-N-acetyl-glucosamine (PNAG) [133]. Immobilized *DspB*-*MagR* showed a high inhibitory effect on biofilm [134]. Using enzymes to degrade polysaccharides that disintegrate biofilm was gradually accepted; the related technology was rapidly expanded. Fan et al. devised a method based on α-amylase to develop a microneedle patch for removing biofilms caused by bacterial infections in wounds (Figure 6c) [135]. Therapeutic strategies of *P. aeruginosa* biofilm infections based on enzyme targeted acidic heteropolysaccharide (Alginate) have been reported [136,137]. Lee et al. cloned an alginate lyase *Aly08* from marine bacterium *Vibrio sp.* SY01 [138]. Daboor et al. also purified alginate lyase *Alyp1400* from marine *P. aeruginosa* [139]. The above extracted lyase could form an efficient combination treatment with antibiotics. Zhang et al. further encapsulated alginate lyase and other drugs to form a silver nanocomposite, and successfully eradicated *P. aeruginosa* infection in the lungs of mice [140]. In addition to alginate, *P. aeruginosa* biofilm also contains polysaccharides *Pel* and *Psl*. Drozd et al. fixed *Pel* hydrolase *PelA* on bacterial cellulose, solving the problem of chronic wound infection [141].

#### 2.2.3. Targeted eDNA Strategy

In 1956, Catlin et al. first observed eDNA as one of the structural components of biofilm, which not only proved that eDNA can be separated from the biofilm matrix but also proved that addition of bovine deoxyribonuclease I (DNase I) can significantly reduce the viscosity of biofilm, eventually leading to diffusion [142]. Subsequent studies have proven that anionic eDNA can chelate cations from the immune system and drugs in the biofilm, providing a “protective umbrella” for bacteria [143,144]. When bacteria are hungry, eDNA acts as a nutrient. In addition, eDNA can also increase the hydrophobicity of the cell membrane, making it easier for bacteria to adhere to the cell surface [145,146]. Thereby, eDNAase synergistic therapy is applied and born [147,148,149]. Based on the above theory, the targeting materials and strategies of eDNA have been put forward successively, and good results have been achieved in removing biofilm and interrupting biofilm formation.

**Figure 7:**
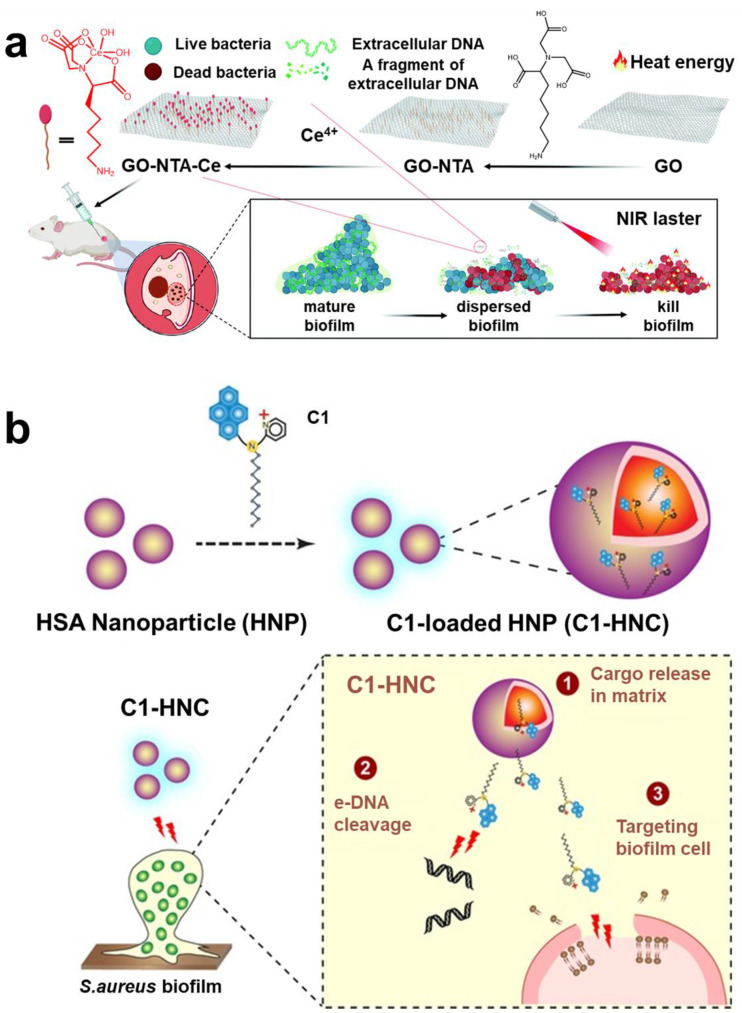
**The eDNA targeting technology.** (**a**) GO-NTA-Ce was used to target and destroy biofilm. Reprinted with permission from Ref. [150]. Copyright 2022, Haiwei Ji. (**b**) Amphiphilic compound C1 with eDNA and membrane-targeting function, assembled with HNP into nanoparticles for targeting and destroying *S. aureus* biofilm. Reprinted with permission from Ref. [153]. Copyright 2016, Prof. Aiyagari Ramesh.

To date, targeted eDNA technology is no longer limited to DNase. Bing et al. designed an eDNAase-simulated artificial enzyme based on graphene-oxide-based naturalistic acid–cerium (IV) composite (GO-NTA-Ce) (Figure 7a) [150]. Qu et al. also designed cerium (IV) complexes (eDNAase mimics) for targeting and hydrolyzing eDNA in biofilm [151]. As the structure and mechanism of eDNA were gradually analyzed, other targeting materials and strategies have emerged. Natural products had always been the first choice for drug research and development. Some natural products with anti-biofilm effects were screened, and it was found that emodin could effectively target eDNA in biofilm [152]. Ramesh et al. reported an amphiphile (C1) with eDNA and membrane targeting, assembling nanoparticles based on human serum albumin for targeting and destroying the biofilm of *S. aureus* (Figure 7b) [153]. Chang et al. screened a fluorescence probe (CDr15), which realized eDNA visualization in *P. aeruginosa* biofilm [154].

#### 2.2.4. Targeted Protein Strategy

**Figure 8:**
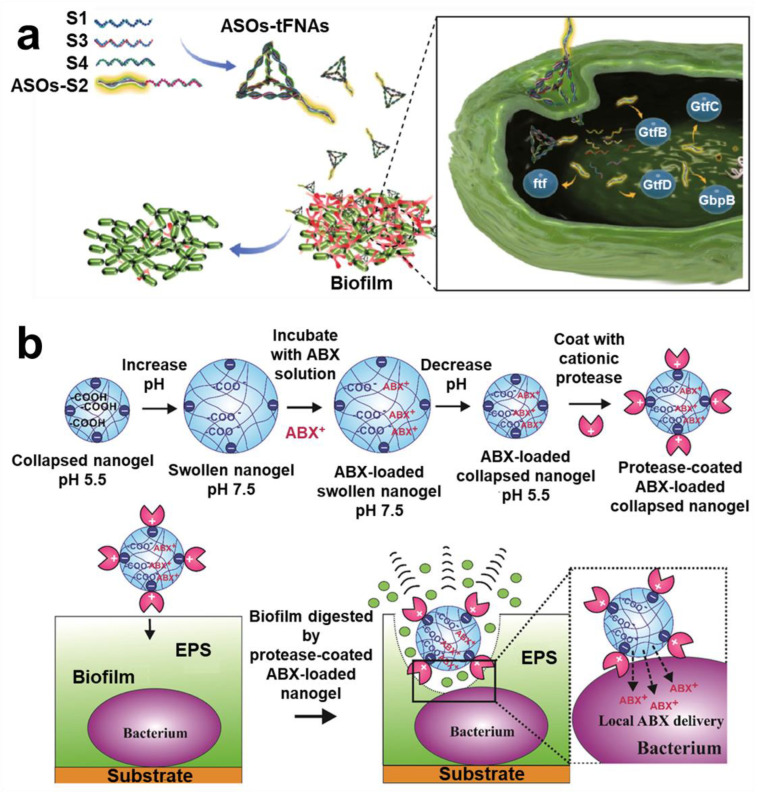
**Protein targeting technology.** (**a**) Scheme of a framework nuclear acid delivery that could target *S. mutans* and destroy biofilm bifunction. Reprinted from Ref. [163]. (**b**) Preparation process and targeting mechanism of gel carrier nanotechnology of protease functionalized. Reprinted with permission from Ref. [165]. Copyright 2019, Vesselin N. Paunov.

Protein plays an important role in promoting formation of biofilm and maintaining structural stability of biofilm [155,156]. More and more evidence shows that biofilm-associated protein can promote development of bacterial biofilm [157,158,159,160]. Interestingly, extracellular proteins do not work alone but jointly with eDNA, polysaccharides, and other components. Some studies have shown that biofilm will spread rapidly after the absence of extracellular proteins in EPS [161,162]. Thus, targeting the protein in biofilm is emerging as a hot research topic. Lin et al. designed a framework nucleic acid delivery that could deliver antisense oligonucleotides to target *S. mutans*, destroying the biofilm (Figure 8a) [163]. The characteristics of carbohydrate–protein interactions were well known. Zhang et al. proposed an inspired nanoplatform composed of spiropyran and galactose. It has dual functions of selectively imaging and eliminating the biofilm in situ [164]. Based on the efficient hydrolysis mechanism of protease to protein, a series of enzyme-functionalized materials were derived. Weldrick et al. introduced a gel carrier nanotechnology based on protease functionality, which, loaded with antibiotics, showed an efficient removal effect on biofilm (Figure 8b) [165]. Devlin introduced that mesoporous silica nanoparticles (MSNs) functionalized by servants could efficiently hydrolyze proteins in *MRSA* biofilm [166]. Curcumin can also target cellular walls and proteins of *Vibrio parahaemolyticus* (*V. parahaemolyticus*) [167].

Numerous compounds with targeting functions have been synthesized and identified to date. Among them, some compounds were effective in reducing substances in biofilm. However, the future research directions of anti-biofilm molecules with targeting function should include several aspects. First, we should consider the species-specific effects of targeted molecules. They may target a substance in the biofilm of pathogenic bacteria, but they may also have the opposite effect on probiotics. In addition, the effect of targeted molecules on normal cell function should be considered. Second, it is worth noting that the targeted molecules are basically targeting a single substance, but it is worth considering whether the targeted molecules will have a cross-reaction effect on the multi-component aspects of biofilm. Third, most studies on targeted molecules are completed in in vitro biofilm models, which means that they are not necessarily applicable to biofilm produced in vivo. Therefore, future research should focus on use of in vivo models to confirm the anti-biofilm activity of targeted molecules.

### 2.3. Targeting Strategy for Biofilm Maturation Stage

In the mature stage of biofilm, bacteria will secrete much EPS and then form a dense mushroom-shaped or pile-shaped mature biofilm with 3D structure [59]. Its internal structure is stable and hydrophobic, which can effectively resist external mechanical forces and drug invasion. Due to the dense encapsulation of EPS, the continuous fermentation, and accumulation of bacterial metabolites in biofilm, a unique chemical gradient microenvironment is formed, such as hypoxia, low pH, negative charge, overexpressed GSH, etc. [168]. These extreme microenvironments will cause some bacteria to enter a dormant and persistent state, thereby reducing the sensitivity of bacteria to antimicrobial agents and antibiotics [169,170]. This is also one of the main reasons why mature biofilm infection is difficult to clear.

#### 2.3.1. Targeted Persistent and Dormant Cells Strategy

After the biofilm is formed, the internal chemical gradient environment of biofilm is hostile to bacteria, so bacteria differentiate into different bacterial subpopulations to protect themselves. In 1942, persistent bacteria were first discovered. They will not develop resistance to drugs, but, because of their slow metabolism, or even dormancy, they can avoid being persecuted by drugs [171]. Similar phenomena have been found in clinical treatment [172]. Therefore, in view of these results, it was proposed that this was equivalent to slow and chronic infection [173]. Targeted dormancy, that is, persistent bacteria, is conducive to removal of biofilm and was more conducive to solving the problems of chronic infection and repeated infection.

**Figure 9:**
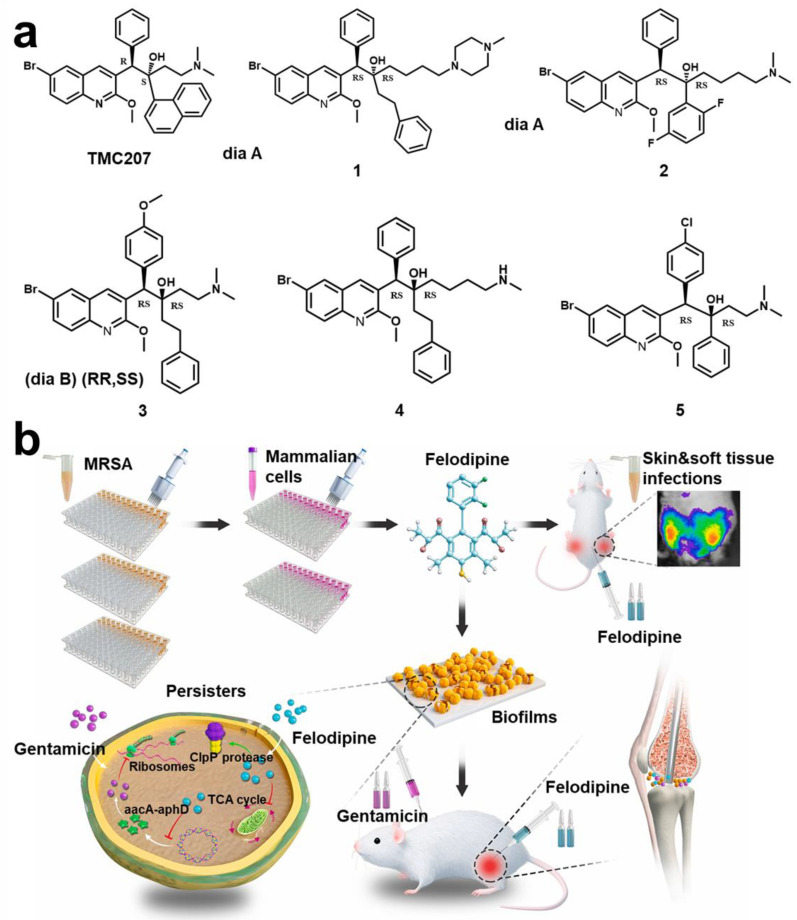
**Persistent and dormant cells targeting technology.** (**a**) Compound structure (TMC207) with the function of targeting persistent bacteria and related derivative structure (Compound **1–5**). Reprinted with permission from Ref. [176]. Copyright 2012, Anil Koul. (**b**) New uses of old drugs using felodipine to regulate bacterial metabolism and improve the clear efficiency of aminoglycosides on persistent cells. Reprinted from Ref. [185].

Typical representatives of dormant bacteria are *Mycobacterium tuberculosis* (*Mtb*). It is reported that targeting persistent bacilli could effectively improve the treatment success rate and shorten the time after granuloma formation [174]. Dialylquinoline TMC207 could target adenosine triphosphate (ATP) synthase, thereby damaging the lipopeptide of the bacterial membrane to achieve the effect of scavenging persistent *Mtb* [175]. Based on structure–activity relationships of TMC207 analogs, many derivative compounds have been gradually reported for targeting persistent bacteria (Figure 9a) [176]. Some researchers also found that halogenated phenazine (HP) derivatives can also effectively target persistent bacteria (*MRSA*; *vancomycin-resistant Enterococcus* (*VER*); *Mtb*) [177]. The stringent response is an adaptive mechanism controlled by response enzyme (*Rel_Mtb_*), which will promote *Mtb* to enter a persistent state. Using lead compound to target *Rel_Mtb_* could directly kill *Mtb* of a dormant state [178]. Some diterpene analytics can also target *Rel_Msm_* and *RelZ* to inhibit formation of persistent cells and biofilm [179]. Narayanan et al. reported that a compound (FNDR-20081) could target maturities *marR* (*Rv0678*, a regulator of *MmpL5*) [180]. Some studies hold that waking up persistent cells is more conducive to killing them than killing them directly [181]. Kim et al. found that adenosine (ADO) can activate ATP and guanosine triphosphate (GTP) synthesis and promote cell respiration, thereby enhancing killing of persistent cells by antibiotics [182]. Rotello et al. proposed a strategy of using biodegradable nanoemulsions to load eugenol and triclosan for synergistic removal of biofilm and persistent cells [183]. In addition, *Acyl* peptide antibiotic ADEP4 is an effective activator of *ClpP* protease, which can adjust persistent *MRSA* [184]. Yue et al. found that felodipine enhanced the clearance efficiency of aminoglycosides on persistent cells (Figure 9b) [185].

#### 2.3.2. The Intelligent Release of Microenvironment Response Strategy

Chemical gradient is one of the classic characteristics of biofilm maturity. Thus far, antibacterial materials that use chemical gradient to achieve intelligent release are constantly emerging. Since this review mainly discusses materials and strategies with targeting function, we will briefly introduce this.

##### Hypoxic

**Figure 10:**
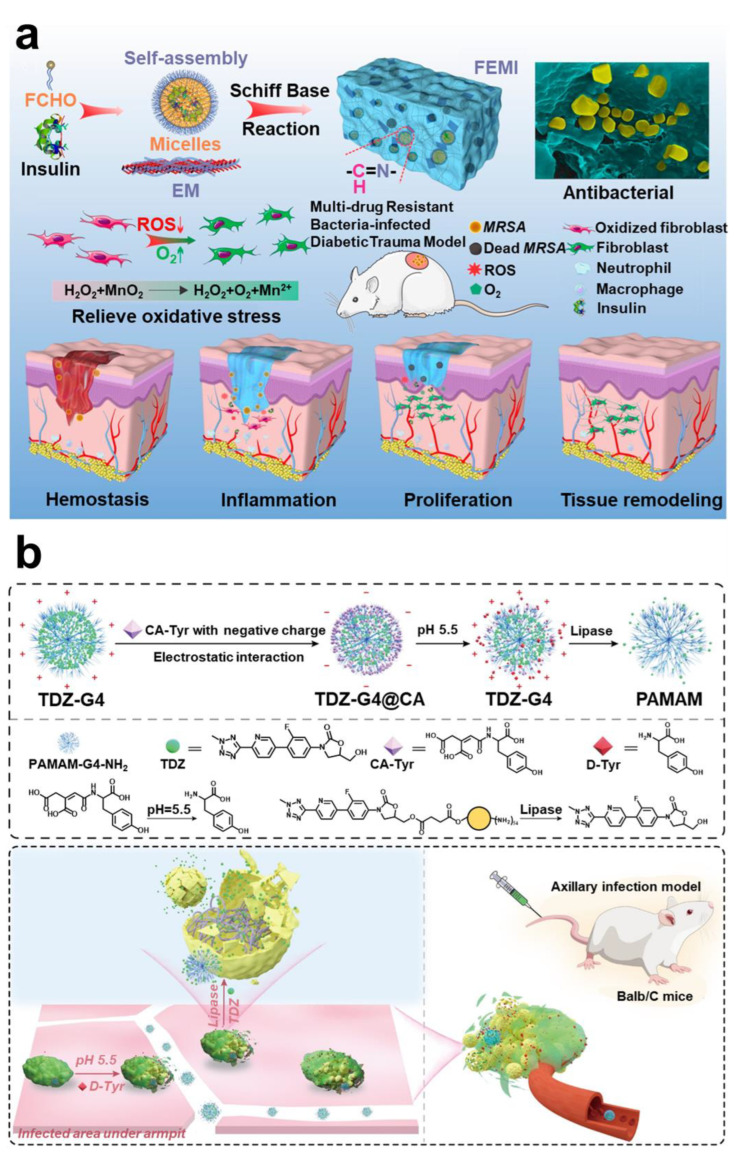
**Using hypoxia and low pH to realize intelligent response technology.** (**a**) Using Mn^2+^ endogenous overexpression of H_2_O_2_ to produce O_2_ to solve the hypoxia. Reprinted with permission from Ref. [194]. Copyright 2020, Qiuyu Zhang. (**b**) Schematic diagram of functional group protonation in low pH environment to realize charge reversal and intelligent release strategy. Reprinted with permission from Ref. [200]. Copyright 2022, Wei Hong.

The hypoxic environment will limit metabolism of bacteria, thereby increasing drug resistance [186]. At the same time, it will also enhance the invasion function and virulence factors of bacteria [187]. Therefore, alleviating the hypoxic environment is an effective method to reverse drug resistance of biofilm. Carrying oxygen (O_2_) can not only effectively overcome a hypoxic microenvironment but also enhance photodynamic therapy (PDT) [188,189,190]. It has also been reported that use of catalysts or enzymes to catalyze the endogenous overexpression of H_2_O_2_ to produce O_2_ can also effectively solve the hypoxic microenvironment of biofilm (Figure 10a) [191,192,193,194,195].

##### Low pH

Lactic acid and acetic acid, which are metabolized by bacteria, will continue to accumulate in the biofilm. At the same time, inflammatory cells continuously release lactic acid, leading to a slight acid phenomenon in the microenvironment of mature biofilm [196,197]. PH-responsive drug delivery systems are widely used in oncology therapy. They are stable in neutral environments but degrade or destroy to release drugs in an acidic environment. Current known degradable bonds that are sensitive to acidity include Schiff bases, esters, ketals, acetals, anhydrides, etc. [198,199]. In addition, using functional groups at a low pH to realize charge reverse and dimensional change is also one of the mainstream strategies in anti-biofilm therapy (Figure 10b) [200,201,202].

##### Negative

**Figure 11:**
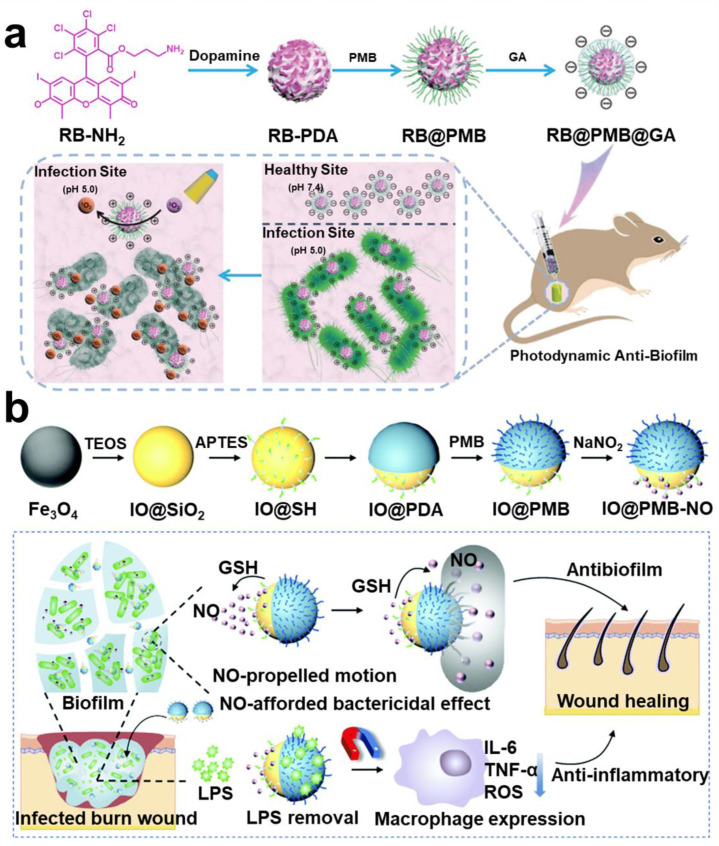
**Using negative overexpression GSH and H_2_O_2_ to realize intelligent response technology.** (**a**) Nanoparticles with charge reversal; the retention capacity of nanoparticles is improved through electrostatic interaction, thereby improving the antibacterial effect. Reprinted with permission from Ref. [206]. Copyright 2021, Fu-Jian Xu. (**b**) A therapeutic regimen that utilizes NO to deplete GSH and trigger immunotherapy. Reprinted with permission from Ref. [210]. Copyright 2022, Xiaohong Li.

The negative microenvironment of mature biofilms is primarily caused by eDNA. The negative microenvironment can effectively neutralize invasion of cationic drugs or antibiotic peptides. Using the negative characteristics to design materials and strategies can enhance penetration and retention of materials into biofilm through electrostatic interaction [203,204]. Strategies that exploit negative features are often combined with other targeting strategies to remove biofilms (Figure 11a) [205,206,207].

##### Overexpression GSH

In biofilm, GSH acts as an antidote against oxidative stress damage to bacteria from reactive oxygen species. In addition, GSH is a major sulfur source for bacteria, and the sulfur metabolic pathway is one of the main causes of bacterial drug resistance [208]. Therefore, using materials to consume GSH in biofilm may make bacteria unable to maintain redox equilibrium, which is favorable for biofilm removal [209]. Some studies have proposed that using endogenous signal molecule nitric oxide (NO) not only consumes GSH but also disintegrates biofilm and promotes immunity (Figure 11b) [210].

##### Hydrogen Peroxide

It is understood that endogenous H_2_O_2_ is over-expressed in the microenvironment of biofilms. As discussed above, H_2_O_2_ is commonly used as a catalytic substrate to produce O_2_ and alleviate a hypoxic microenvironment. H_2_O_2_ is converted into toxic hydroxyl radicals and superoxide radicals under catalysis of peroxidase (POD) or catalyst [211,212]. This kind of treatment is called chemokinetic therapy (CDT) [213]. This method does not cause bacteria to become resistant.

#### 2.3.3. Other Targeting Strategies

For mature biofilms, in addition to the targeted strategies reviewed above, there are different technical targeting strategies that can still be effective in eradicating biofilms. Rapid developments in biotechnology, nanotechnology, and chemical engineering provide unparalleled flexibility for anti-biofilm technology. Functionalized nanoparticles offer the advantages of controllable structure, morphology, charge, size, target, and optional antibacterial methods. These nanostructures can be used to accurately target and clear the biofilms while avoiding bacterial resistance. We focus on the overall concept and review some nano research in vivo models with clinical potential.

##### Magnetic Targeting Technology

**Figure 12:**
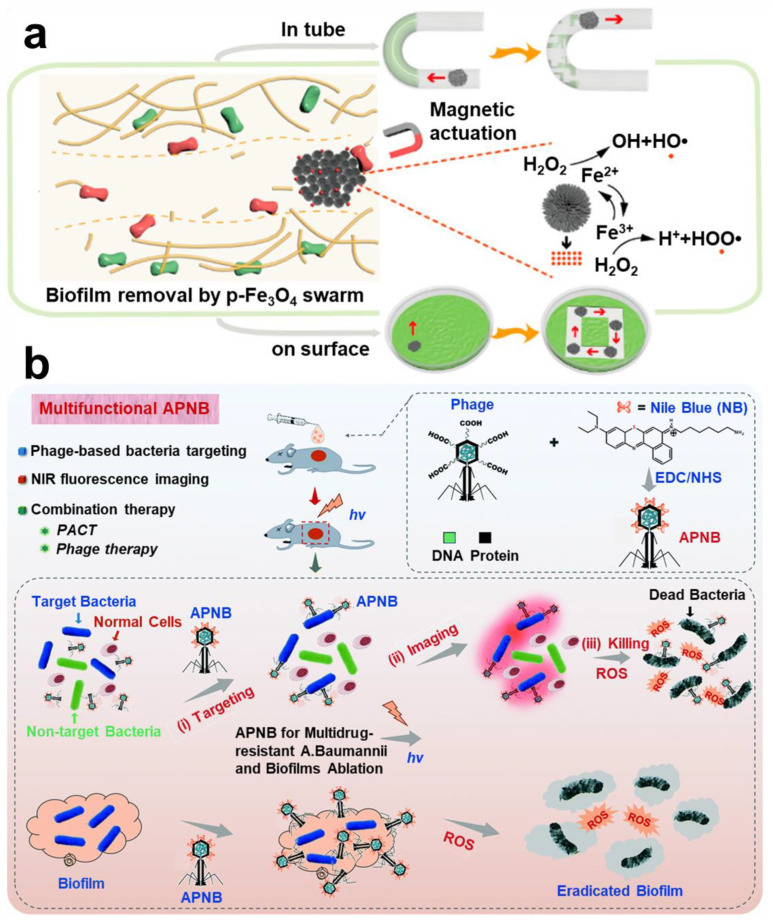
**Other targeting strategies in anti-biofilm infection.** (**a**) Magnetic targeting nanoparticles prepared by Fe_3_O_4_ and the Fenton reaction antibacterial mechanism. Reprinted with permission from Ref. [215]. Copyright 2021, Li Zhang. (**b**) Therapeutic scheme for eradicating biofilm using phage-targeting technology and photodynamic therapy. Reprinted from Ref. [224].

Iron-based nanoparticles have stable paramagnetic properties. Among them, Fe_3_O_4_, which is tether-free and harmless to the human body, has been widely applied in construction of *magnetic micro-robots* [214]. Meanwhile, Fe_3_O_4_ may promote the Fenton reaction, which has certain antibacterial properties. Zhang et al. designed a magnetic micro swarm based on porous Fe_3_O_4_ masterclass, which showed efficient removal of biofilm (Figure 12a) [215]. Shi et al. loaded glucose-oxidase and L-arginine on Fe_3_O_4_@SiO_2_ to deliver nanoparticles to the infected site in mice by magnetic targeting technique. Nanoparticles achieve a cascade reaction to produce NO to eliminate the biofilm infection of drug-resistant bacteria [216]. Escarpa et al. reported a dual-propelled (both catalytic and magnetic) lanbiotic-based Janus micromotor, which can efficiently and selectively capture/inactivate Gram-positive bacteria and biofilms [217].

##### Phage-Targeting

Bacteriophage–bacteria interaction has been a hot topic and research frontier. Specific targeted function of bacteriophages has been used in most therapeutic areas, such as intestinal infection, intracellular bacterial infection, and liver disease [218,219,220]. As a result, *phage-targeting techniques* have also appeared in treatment of biofilm infection. Yang et al. designed a strategy of combining phage-guided targeting with AIEgens photodynamic inactivation (PDI) [221]. Sharma et al. found a bacteriophage targeting drug resistance *Enterococcus faecalis* (*E. faecalis*) biofilm; it is worth noting that this phage can be administered orally [222]. Hazan et al. also screened a phage targeting *E. faecalis* biofilm [223]. Wang et al. reported a bacteriophage-photodynamic antibacterial chemotherapy for precise antibacterial and biofilm ablation (Figure 12b) [224]. Hatful et al. reported for the first time the therapeutic effect of bacteriophages on multi-drug-resistant *Mycobacterium chelonae* and described the observed clinical efficacy. The results suggest that bacteriophages are a promising treatment. However, the safety of phage therapy needs to be investigated further [225].

##### Probiotic Targeting

Since the introduction of probiotic targeted delivery, it has been widely used in a variety of fields, including improving gut flora, oncology, and immunotherapy. In addition, *probiotic delivery techniques* have been widely used in anti-infection applications. This technique not only disintegrates biofilms of pathogenic bacteria but also effectively stimulates the immune system, resulting in a distinct antibacterial–immune combination treatment regimen. Chapman et al. found that four probiotics (*Lactobacillus acidophilus* NCIMB 30184 (PXN 35); *Limosilactobacillus fermentum* NCIMB 30226 (PXN 44); *Lactiplantibacillus plantarum* NCIMB 30187 (PXN 47); and *Lacticaseibacillus rhamnosus* NCIMB 30188 (PXN 54)) could inhibit biofilm formation of pathogenic bacteria through competing for binding sites on the host bladder epithelium, and adhesion of urinary tract pathogens was inhibited [226]. Lorenzo Drago et al. observed two probiotics (*Streptococcus salivarius* 24smb and *S. oralis* 89A) could inhibit biofilm formation of specific pathogens and even disperse their preformed biofilm [227]. Gabriele Meroniet et al. summarized that lactic acid bacteria could inhibit the role of pathogenic bacteria biofilm through multiple pathways [228]. Successive studies of probiotics against pathogenic bacteria have shown that probiotics have the function of targeting and inhibiting disease-causing bacteria pathogenic bacteria and have great potential as drugs or drug vectors [229,230,231].

##### Gene Targeting

*Gene targeting techniques* alter endogenous genes of bacteria by homologous recombination. The effects of this targeting technology could be lasting. In addition to the advantages of directly disintegrating biofilms, it may also directly shadow the dormant cells or newly dividing cells, leading to unique therapeutic effects [232]. Thorsten M. Seyler et al. reported a derivative of PKZ18 (PKZ18-22) for the first time, which can selectively target Gram-positive bacteria [233]. CRISPR interference (CRISPRi) was also one of the main technologies developed in the field of anti-infection [234]. Kimberly A. Kline et al. developed a dual-carrier nisin-inducible CRISPRi system in *E. faecalis* that can target and effectively silence resistance genes via non-template and template chains [235]. In addition, numerous *gene targeting techniques* have been applied in the research and development of antimicrobial drugs [236,237,238].

##### Metabolic Targeting

The metabolic pathway of drug resistance has consistently been one of the hotspots of antimicrobial research. It has the potential to reduce bacterial resistance or restore bacterial sensitivity to antimicrobials in a number of ways. Shatalin et al. designed a cystathionine based on bacterial hydrogen sulfide (H_2_S) to increase antibiotic resistance *γ-Lyase* (CSE) inhibitor. The inhibitor takes CSE as its target, which inhibits production of H_2_S and reduces antibiotic resistance [208]. Other studies have also shown that targeting drug resistance genes can be used to develop new antibacterial drugs [64,239].

Mature biofilms are the model used in most laboratory studies, so there are a myriad of fascinating targeting techniques available at this stage. These studies provide a valuable theoretical basis for clinical transformation, and even some targeting techniques can target specific cell subsets in multi-strain biofilm. The wide development of biofilm-targeting technology should consider the following points: first, biological effects of materials between host and bacteria; second, the whereabouts and potential hazards for host of the materials after antibacterial processes in vivo. The most important point is whether the targeted material has a negative effect on the normal flora during or after treatment.

### 2.4. Targeted Strategy for Biofilm Dispersion Stage

The biofilm dispersal phase is a unique phase that represented the transition from bacterial biofilms to planktonic bacteria and represented the final step in the biofilm life cycle. Dispersed planktonic bacteria lose their “shelter” and “umbrella”, so they are easier to kill. As a result, some studies have considered active dispersal of biofilms as a promising method to control biofilm removal [240]. However, several studies have considered active dispersal of biofilms as a promising method to control biofilm removal. The biofilm should be prevented from entering the dispersion stage [241,242,243]. Therefore, in this chapter, we discuss the application of targeting technology from two parts: voluntary dispersion biofilm and limited biofilm dispersion.

#### 2.4.1. Active Dispersion Biofilm

**Figure 13:**
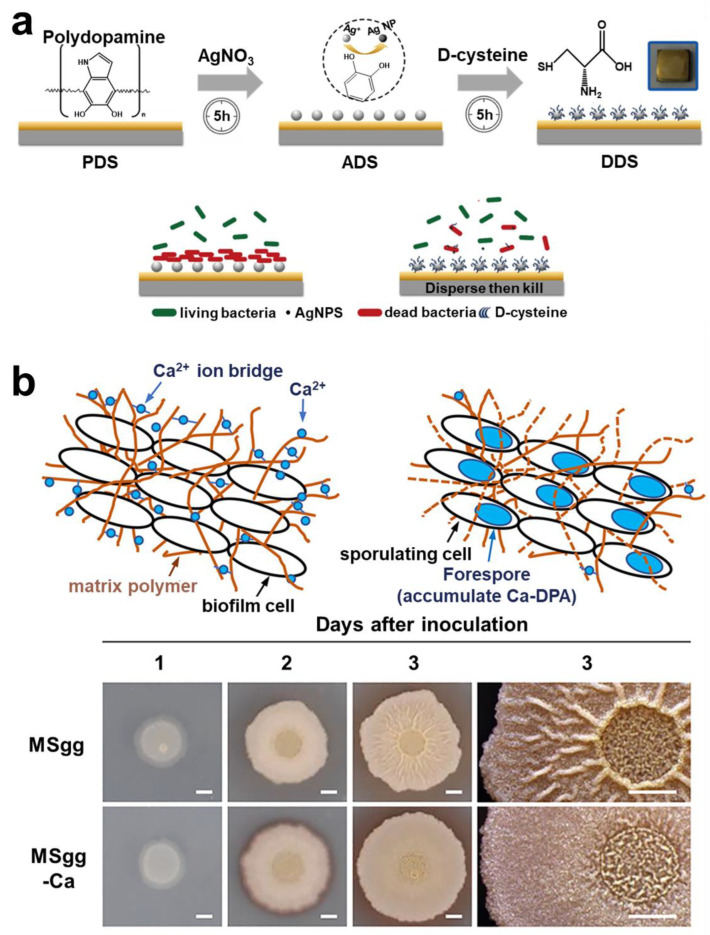
**Targeting technology and regulation technology of biofilm dispersion stage.** (**a**) The therapeutic strategy of using D-type amino acids to achieve antibacterial and dispersive functions. Reprinted with permission from Ref. [249]. Copyright 2020, Dawei Zhang. (**b**) The strategy of using calcium ions to regulate the biofilm and prevent it from entering the dispersion stage. Reprinted with permission from Ref. [253]. Copyright 2021, Kazuo Kobayashi.

After the biofilm has grown to a certain size, the bacteria will actively disrupt the biofilm, thus achieving diffusion. Currently, most studies have proposed various strategies for dispersing biofilm based on the mechanism of bacterial self-degradation of EPS. As the strategy of enzymatic hydrolysis of EPS has been fully discussed above, the discussion will not be repeated in this section. D-type amino acid is one of the main compounds secreted by bacteria in the biofilm dispersion stage [244]. Therefore, therapeutic strategies have been proposed to combine D-type amino acids with drugs. Part of the D-type amino acids was initially used to label peptidoglycan of bacteria, thus achieving effective targeting. However, some D-type amino acids can efficiently cleave EPS in biofilms. Interestingly, this cleavage effect was only directed at the bacterial biofilm and is harmless to normal cells. Cláudia et al. constructed a nanoparticle functionalized with D-amino acids, which can break down the biofilm, thereby improving the bactericidal effect of moxifloxacin in the biofilm [245]. Simple antibiotic-D-amino acid combination therapy could also effectively eradicate biofilm infection of drug-resistant bacteria [246]. Wang et al. constructed a chiral-glutamate-functionalized gold nano bipyramid (Au NBP). The results showed that D-Glu-Au NBPs could more accurately target bacterial cell walls and eliminate biofilms [247]. Li et al. designed a kind of micelle, and the D-Tyrosine loaded on the micelle was released in an acidic environment to decompose the biofilm matrix [248]. Most studies have demonstrated the great clinical value of D-type amino acid, a dispersal factor of bacteria (Figure 13a) [249,250]. Furthermore, Olivier et al. first studied the effect of human hormone atrial natriuretic peptide (hANP) on formation and dispersion of *P. aeruginosa* biofilm [251].

#### 2.4.2. Control Biofilm Dispersion

Some researchers believe that the control of biofilm dispersion is significant compared to active dispersive biofilm techniques. The main reason for this is that the control of biofilm dispersion can be manually controlled both spatially and temporally. Moreover, it can effectively address the problem of secondary infection of biofilm residues. Manju et al. showed that *RV_1717_* was a kind of β-D-galactosidase in the cell wall. It has been demonstrated experimentally that *RV_1717_* expression is downregulated, which prevents *Mtb* from dispersing from the biofilm in vitro [252]. Kobayashi et al. found that adding Ca^2+^ to the culture medium could counteract the biofilm dispersion mechanism in the study (Figure 13b) [253]. Although the research on regulating of biofilm dispersion is relatively limited, it provides a fresh theoretical basis for development of new drugs.

## 3. Conclusions and Perspectives for Future Research

Tremendous development in bacterial targeting technology has occurred in recent years, including *metabolic targeting*, *gene targeting*, *membrane targeting*, *protein targeting*, and *extracellular matrix targeting*. Compared to conventional antibacterial materials, the targeting technique is more accurate and efficient and, therefore, has better antibacterial properties and ablation efficiency for biofilm. The intensive study of biofilm has greatly accelerated the pace of biofilm-targeting technologies. Targeting techniques have significantly improved biocompatibility by fine-tuning the life-cycle properties of biofilms and related components, combined with diagnostic imaging techniques to unlock high-dimensional multimodal studies. Based on these advantages, biofilm-targeting technology has been applied to ultra-sensitive diagnosis and personalized treatment. This paper reviews the known biofilm-targeting technologies, with a particular focus on targeting dormant cells and the regulation strategy for biofilm life cycle. While significant progress has been made at this stage, as described in this paper, there are still many challenges in clinical targeting technology:

1. The key barrier is the in vivo biofilm model, especially for a multi-species biofilm model. In this regard, substantive research on the targeted techniques should be conducted before entering the clinic; it is extremely important to implement techniques that can accurately target the objective in multi-species biofilm.
2. Further implementation of differential targeting of pathogenic bacteria and probiotics is highly beneficial and is expected to facilitate rapid development of immunotherapies.
3. To clarify the metabolic pathway of targeted techniques under host pathological conditions, it is necessary to develop targeted techniques with long-term visualization or monitoring.
4. Currently, targeting techniques target different phases of biofilms. Could there be a technique to observe the biofilm phase in patients to make treatment plans more effective?
5. The biological effects of targeting technology among materials, cells, and bacteria are very worthy of study.
6. Currently, small molecules of targeted inhibitors have the potential to replace antibiotics for treatment, but antibiotics have a chiral structure. Research on the combination of targeted inhibitors and stereochemistry may be a new generation of antibiotic research and development route.
7. Targeting technology is needed to meet clinical needs. Cost-effective, simplified, and economical amplification preparation strategies need to be widely studied.

In the rapidly evolving antibacterial field, we assume that continuous improvement in biofilm-targeting technology will make it possible to target in an accurate way and introduce single-bacterial targeting technology that is not available at present. This is not only conducive to accurate clinical diagnosis and treatment but also helps to stimulate discovery of new technologies.

## Figures and Tables

**Table 1:** Characteristics, targets, and targeting advantages of biofilm at different stages.

Biofilm Types	Characteristic	Target	Pros	Cons
Initial adhesion stage	Reversible and Irreversible adhesion.	Adhesin and Adhesion protein.	Prevention preferable to treatment.	Stability of surface coatings.
			Will not cause drug resistance.	Not necessarily kill bacteria.
			Access not required after implantation.	Potential substrate utilization by host.
Early formation stage	Active intercellular Communication and progressive formation of EPS.	QS; Polysaccharide Intracellular Adhesin (PIA); eDNA; Polysaccharides and Proteins.	Molecular medicine.	Potential degradation by nucleases, proteins, or enzyme.
			Controlled locally.	Highly localized.
			It will affect metabolism and will not produce drug resistance.	Composition variability.
Maturation stage	Mature EPS and Gradient chemical microenvironment and Changes in bacterial metabolism.	Hypoxic; Low pH; Negative; Overexpression GSH; H_2_O_2_; Persistent and dormant bacteria.	Disrupt pathogenic microenvironment.	Difficult to simulate in vivo models.
			Readily functionalized.	Incomplete eradication.
			Active on dormant cells.	Interaction with host.
Dispersion stage	Accumulation of biofilm residues and associated secretions.	Enzymes, D-amino acids; surfactants and others.	Readily combined with antimicrobials.	Low spatiotemporal controllability.
			Avoid cell dormancy.	Residues to be resolved.
			High universality.	Release of pathogens may result in recolonization and acute infection.

## Data Availability

Data sharing not applicable.
